# Assessing the Role of Daptomycin as Antibiotic Therapy for Staphylococcal Prosthetic Joint Infection

**DOI:** 10.7150/jbji.41278

**Published:** 2020-03-30

**Authors:** Alberto V. Carli, Andy O. Miller, Milan Kapadia, Yu-fen Chiu, Geoffrey H. Westrich, Barry D. Brause, Michael W. Henry

**Affiliations:** 1 Hospital for Special Surgery, Division of Adult Reconstruction & Joint Replacement, 535 East 70th Street, New York, NY 10065, USA; 2 Hospital for Special Surgery, Infectious Disease Division, 535 East 70th Street, New York, NY 10065, USA

**Keywords:** prosthetic joint infection, daptomycin, antibiotics, revision arthroplasty, implant retention, staphylococcal

## Abstract

**Background**: The role of daptomycin, a potent, safe, convenient anti-staphylococcal antibiotic, in treatment of prosthetic joint infection (PJI) is unclear. We evaluated our experience with the largest cohort of patients with staphylococcal PJI managed with daptomycin.

**Methods**: A cohort of staphylococcal hip and knee PJI treated with daptomycin was identified by hospital records from 2009 to 2016. All cases met Musculoskeletal Infection Society International Consensus criteria for PJI. The primary endpoint was 2 year prosthesis retention. Univariate analyses and regression statistics were calculated.

**Results**: 341 patients with staphylococcal PJI were analyzed. 154 two-stages (77%) and 74 DAIR procedures (52%) met criteria for treatment success at 2 years. 77 patients were treated with daptomycin, of which 34 two-stages (68%) and 15 DAIRs (56%) achieved treatment success. Pairwise and regression analysis found no association between treatment success and daptomycin use. Organism (DAIR only) and Charlson Comorbidity Index scores (DAIR and two-stage) were significantly associated with treatment outcome. Six daptomycin patients (7.8%) had adverse side effects.

**Discussion**: Daptomycin fared no better or worse than comparable antibiotics in a retrospective cohort of staphylococcal hip and knee PJI patients, regardless of surgical strategy.

**Conclusion**: The convenient dosing, safety, and potency of daptomycin make it an attractive antibiotic for staphylococcal PJI. However, these advantages must be weighed against higher costs and rare, but serious side effects.

## Introduction

Periprosthetic joint infection (PJI) following total joint replacement surgery is a devastating complication that is associated with poor functional outcomes^1^ and a higher risk of all-cause mortality^2^. Staphylococci, the most common PJI pathogens, access the periprosthetic space either during the perioperative period or through hematogenous dissemination^3^. Recent reports suggest that antibiotic- resistant strains are becoming more common^4^,^5^.

Standard parenteral antibiotics for methicillin- susceptible staphylococcal PJI generally antistaphylococcal penicillins and cephalosporins, such as oxacillin and cefazolin respectively, Vancomycin and teicoplanin (outside of North America) are glycopeptide antimicrobials used to treat methicillin-resistant staphylococcal PJI. All of these medications have notable limitations in efficacy, and have not been demonstrated to reliably sterilize retained orthopedic implants in laboratory or clinical settings. Resistance to teicoplanin has been documented to occur in up to 9% of staphylococcal PJI^4^ and nephrotoxicity has been associated both with systemic use and (less commonly) within antibiotic cement spacers^6^,^7^. Furthermore, vancomycin requires careful dose titration which can be difficult in non-acute care settings.

An alternative to the above antibiotics is daptomycin, a cyclic lipopeptide that has selective activity against aerobic, anaerobic, and facultative gram-positive bacteria. Daptomycin coordinates with calcium ions and bacterial phospholipids to depolarize bacterial cell membranes, producing a rapid bactericidal effect^8^. Daptomycin has a simplified dosing schedule, has few drug-drug interactions due its lack of metabolism by cytochrome P450 or other hepatic enzymes^9^, and is far less nephrotoxic than vancomycin^10^. Daptomycin is active against most MRSA. There is limited evidence that daptomycin may improve outcomes in MRSA infections, relative to vancomycin monotherapy. For instance, in a matched cohort of 262 patients with MRSA bacteremia, daptomycin was associated with less clinical failure and lower mortality^11^. *In vitro*^12^, and in animal models^13^-^15^, daptomycin shows better ability than vancomycin to kill MRSA in biofilms, particularly when combined with rifampin. Despite its higher cost, daptomycin may remain cost-effective in treating systemic MRSA infections^16^,^17^; since the introduction of generic daptomycin in 2016, acquisition costs have decreased significantly.

To date, small series of fewer than twenty consecutive PJI patients receiving daptomycin as their primary treatment antibiotic have been published. A recent paper noted no differences in outcome, but an increased safety profile, in patients receiving daptomycin (versus vancomycin) for empiric postoperative antibiotic therapy; in this study empiric daptomycin was given for only 6 days^18^. No larger studies have compared definitive treatment with daptomycin versus other antimicrobials across a single cohort. Chang^19^ evaluated 16 PJI patients who could not receive vancomycin therapy either due to bacterial resistance, previous treatment failure with vancomycin, or stage 3 or 4 renal disease. They found that treatment success when combined with surgical treatment was 87.5% with no severe side effects reported.

Due to its antimicrobial profile and documented rapid penetration into bone and synovial tissue during joint replacement surgery^20^, daptomycin may have a promising role in the treatment of PJI. We therefore present our experience with daptomycin in the treatment of both acute and chronic staphylococcal PJI with minimum 2-year follow-up.

## Methods

Institutional Review Board approval was obtained prior to study. We queried a retrospectively gathered periprosthetic joint infection database in our tertiary orthopedic institution to identify all total hip arthroplasty (THA) and total knee arthroplasty (TKA) cases with staphylococcal PJI from January 2009 to February 2016. To accomplish this, hip and knee patients with an ICD-9-CM code of 996.66 or ICD-10-CM codes of T84.51*, T84.52*, T84.53*, T84.54* for PJI were first identified. These patients were then cross-referenced with a subset of ICD-9-PCS or ICD-10-PCS codes and CPT billing codes to identify those who underwent either implant removal in the setting of a two-stage revision or a debridement with implant retention (DAIR). All qualifying cases then underwent detailed chart review to include only those that met Musculoskeletal Infection Society (MSIS) diagnostic criteria for PJI prior to treatment^21^. Staphylococcal PJI was defined as at least one deep tissue culture growing a microorganism from the genus *Staphylococcus*. Acute PJI was defined as a PJI diagnosed fewer than 90 days after arthroplasty.

Through chart review, patient demographics including height, weight, preoperative lab values within two weeks of surgery, comorbidities, prior arthroplasty history, clinical presentation and symptoms were collected. Charlson comorbidity index (CCI) scores^22^ were calculated for each patient and then categorized into groups of score of 0, 1-2, and ≥ 3. Diagnostic criteria including serum and synovial inflammatory markers, findings from imaging studies, surgical procedure details and all microbiology results were collected. All patients were assessed by a board-certified infectious disease specialist and managed by a multidisciplinary team during admission. Intraoperatively, 5 or more joint tissue samples were collected and intraoperative antibiotics were held prior to culture collection. Patients underwent implant removal or DAIR according to surgeon preference and clinical indications. For DAIR, the volume of irrigation, use of antiseptics and exchange of modular components was left to the discretion of the surgeon. When implants were removed, a polymethylmethacrylate (PMMA) spacer containing vancomycin and an aminoglycoside (either gentamicin or tobramycin) was placed. The type of PMMA used and the dosage of antibiotic were left to the discretion of the surgeon.

Following surgery, all patients received intravenous antibiotic therapy until postoperative day 42. Vancomycin and a broad-spectrum beta-lactam agent were generally given empirically immediately following surgery, and then antibiotics were adjusted according to microbiologic results. Antibiotic regimens were selected in a non-randomized multifactorial way as follows: while most patients with methicillin-sensitive staphylococcal PJI received anti- staphylococcal beta-lactams (cefazolin or oxacillin), some were treated with daptomycin because of allergy or to provide a convenient home dosing schedule. Among patients with methicillin- resistant pathogens, daptomycin or vancomycin were selected based on a variety of factors including: evidence of higher risk of vancomycin-induced kidney injury, ability to tolerate and participate in vancomycin dose titration at home, ability to achieve therapeutic levels of vancomycin at home, insurance coverage considerations for daptomycin, and possible potency differences of daptomycin versus vancomycin in MRSA with vancomycin MIC > 1 ng/microliter. In addition, daptomycin was occasionally started in cases of vancomycin nephrotoxicity or other intolerance. Data on daptomycin dose was not captured for many patients in this cohort. Daptomycin was dosed between 6 and 9 mg/kg intravenously once daily, often rounded up to the nearest 250mg. No patient received daptomycin at a dose of more than 9 mg/kg.

Following hospital discharge, antibiotic therapy was provided until postoperative day 42 (6 weeks in total) via an outpatient infusion pharmacy, an outpatient infusion center, or an inpatient rehabilitation (although such facilities rarely provided daptomycin due to economic constraints). Standard blood tests were performed weekly to assess for signs of nephrotoxicity, hepatotoxicity, myelotoxicity, and in the case of daptomycin, myotoxicity as well; vancomycin trough levels were monitored at least once per week. For DAIR cases, intravenous antibiotic therapy was combined with oral rifampin therapy (at a daily dose of 600mg), and was followed by oral antibiotic suppression that continued for a minimum of 3 months. Rifampin was provided for no less than 6 weeks, and no more than 6 months.

For two-stage exchange, a minimum 2-week antibiotic holiday preceded reimplantation, often but not always confirmed with preoperative aspiration or intraoperative frozen sections, and apart from 24 hours of perioperative prophylaxis, antibiotics were not routinely prescribed postoperatively.

For PJI cases treated with DAIR, success was defined as prosthesis retention at 2-year follow up after the last surgical procedure. Therefore, patients who underwent multiple DAIR procedures within the same admission, but still retained their original non- modular components 2 years later, were considered successful. For patients treated with two-stage exchange, success was defined as retention of the newly implanted prosthesis with no further surgical procedures secondary to infection in the two years following reimplantation^23^. Clinical outcome was obtained through chart review of follow up visits with either the orthopedic surgeon or infectious disease specialist.

### Statistics

Continuous variables such as BMI and age are presented as the mean and standard deviation (SD) and were compared using a Student t test or Mann-Whitney U test. Categorical variables such as sex, parenteral antibiotics and intervention performed are presented as frequencies and were compared using the chi-square test or the Fisher exact test. Logistic regression analyses were performed to identify risk factors associated with treatment outcome. The sample population was normally distributed. All tests were 2-sided. Significance was defined as p < 0.05. Kaplan-Meier survivorship curves were generated for 2-year follow-up with 95% confidence interval (CI). Statistical analyses were performed using SAS 9.4 (SAS Institute Inc., Cary, NC).

## Results

341 patients met inclusion criteria for staphylococcal PJI within the study period. 158 patients were infected with methicillin sensitive *Staphylococcus aureus* (MSSA), 60 with MRSA and 123 with coagulase-negative *Staphylococcus* (CONS). 77 patients (22.6%) were treated with daptomycin. There was no significant difference in patient demographics, patient comorbidities or singular bacterial species between daptomycin and non-daptomycin groups [Table 1]. The overall success rate of staphylococcal PJI treatment was 66% and success rate of staphylococcal PJI treated with daptomycin was 64%.

With regard to treatment success, two-stage exchange patients had a significantly increased rate of treatment success (154 of 200, 77%) compared to DAIR (74 of 141, 52%; p<0.0001) at 2 years. In multivariate regression, bacterial species were found to be significantly associated with treatment outcome only in the DAIR group, with CONS (odds ratio=5.58, p = 0.007) and MSSA (odds ratio=3.68, p=0.031 respectively) yielding significantly better rates of treatment success rates compared to MRSA [Table 2]. CCI scores were significantly associated with treatment outcomes in both DAIR and two-stage groups, with CCI scores ≥3 being associated with significantly higher treatment failure rates in both pairwise comparisons (p=0.009, p=0.012, respectively) and regression analysis (p=0.003, 0=0.014, respectively).

Treatment success with two-stage use did not significantly differ in daptomycin (34 of 50, 68%) versus non-daptomycin groups (118 of 150, 79%; p=0.126) [Table 3]. Treatment success with DAIR did not significantly differ in daptomycin (15 of 27, 56%) versus non-daptomycin groups (59 of 114, 52%; p=0.72) [Table 3]. When holding patient demographics, and bacterial species constant, treatment with daptomycin was not significantly associated with DAIR or two-stage treatment success (p=0.170, p=0.301, respectively) [Table 2]. For patients who did not achieve treatment success with daptomycin, no daptomycin-resistant strains were detected in subsequent cultures.

**Figure 1:**
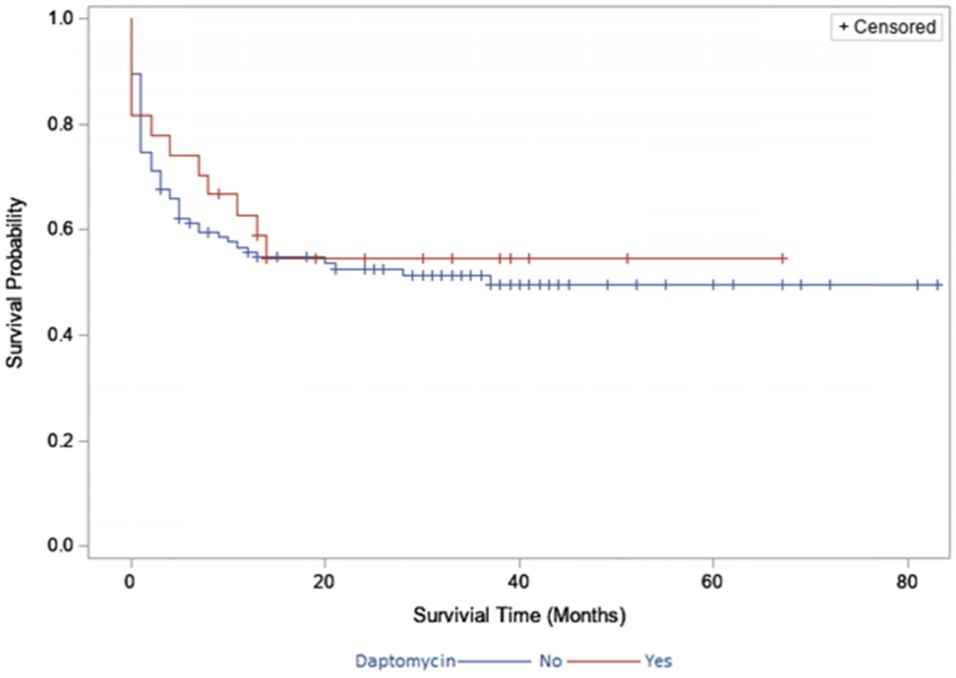
Survivorship in the debridement, antibiotics and implant retention group stratified by daptomycin use.

**Figure 2:**
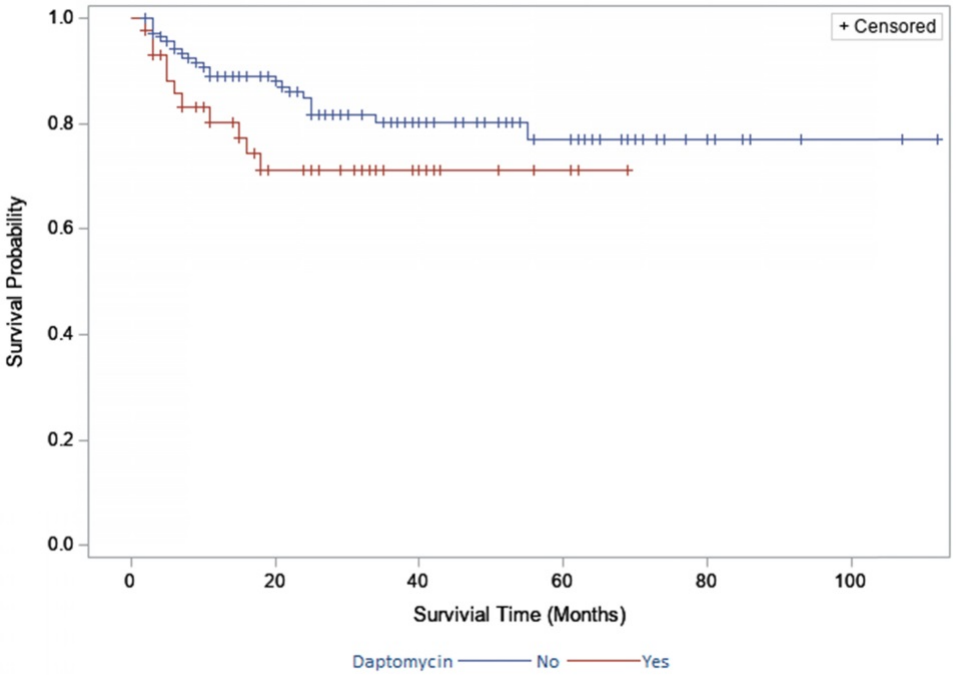
Survivorship in the two-stage exchange group stratified by daptomycin use.

The two-year survivorship for DAIR patients treated with daptomycin was 55% (95% CI, 36%-74%; Fig. 1) and 53% (95% CI, 43%-62%; Fig. 1) for non- daptomycin patients. The two year survivorship for two-stage exchange patients treated with daptomycin was 71% (95% CI, 56%-86%; Fig. 2) and 85% (95% CI, 78%-91%; Fig. 2) for non-daptomycin patients. There were no significant differences in survivorship when stratified by daptomycin use in the DAIR group (p=0.711) or two-stage exchange group (p=0.121).

Six patients (7.8%) had complications associated with daptomycin use. Two patients that had undergone two-stage exchange followed by daptomycin treatment were diagnosed with eosinophilic pneumonitis, one occurring at 6 days and the other 40 days after commencement of therapy. Both cases resolved following cessation of daptomycin. Furthermore, four patients exhibited serum elevations of creatine phosphokinase and two were subsequently diagnosed with rhabdomyolysis within two weeks of treatment, prompting cessation of daptomycin and rapid recovery. One patient in the daptomycin group that underwent a two-stage exchange died three days following explantation due to cardiac arrest, but daptomycin was not considered to be associated with the mortality.

## Discussion

This study represents the single largest cohort of PJI patients treated with daptomycin to date. In our series, daptomycin treatment following DAIR or two-exchange revision was no more or less effective than comparable antibiotics. Daptomycin was tolerated well in 92% of our patient cohort: the convenient dosing, lack of nephrotoxic risk, and putative enhanced anti-staphylococcal potency of daptomycin make it an appealing choice for postoperative antibiotic therapy in PJI. However, these advantages must be carefully weighed against higher costs and occasional serious side effects.

Two patients in our cohort developed eosinophilic pneumonitis, an unusual and poorly- understood complication associated with daptomycin. The largest study to date of complications secondary to daptomycin administration found, in 11,557 patients, an estimated incidence of eosinophilic pneumonitis of 0.03% 24. A review of the US FDA Adverse Event Reporting System database identified 7 definite, 13 probable, and 38 possible cases of daptomycin-induced eosinophilic pneumonia^25^. A recent literature search in PubMed confirmed 32 case reports meeting formal criteria for eosinophilic pneumonitis, with most resolving following cessation of daptomycin. Given the rarity of published reports of this unusual pulmonary entity, it may be underdiagnosed. Further study is required to identify risk factors for eosinophilic pneumonitis and to best diagnose it the PJI cohort.

Four patients in our daptomycin cohort exhibited increases in serum creatine phosphokinase levels, with two developing clinical rhabdomyolysis. The association between daptomycin use and muscle damage has been well demonstrated: phase III clinical trials reported a 2.8% incidence of elevated CPK levels and 0.2% incidence of myopathy^26^. The mechanism of action has been described *in vitro* and is associated with daptomycin-induced interruption of myocyte membrane electrical potentials, leading to inhibition of muscle contractions and pain^27^. Regular and prompt serum measurement of creatine phosphokinase (CPK) is recommended^28^. Rhabdomyolysis remains a rare complication of daptomycin therapy as long as daptomycin is infused every 24 hours and CPK is monitored regularly.

We acknowledge limitations in this study. Daptomycin use was often subject to patient insurance coverage, and although we did not identify any difference between patients who received and did not receive daptomycin in demographic data, pathogen, or surgical strategy, we acknowledge that possible selection bias or confounding effects are possible. Furthermore, the surgical indications for DAIR and two-stage revision were not always clearly defined, making surgical treatment a potential confounder in determining why treatment failed. DAIR procedures were not standardized by type and volume of irrigation utilized. Similarly, the type of cement and dosage of antibiotic in two-stage revisions was not standardized, nor were the diagnostic criteria for confirming eradication and clearance for reimplantation. Although the lack of treatment standardization in PJI is not unique to our study^29^-^32^, we acknowledge that this makes interpretation of a single variable (the intravenous antibiotic) difficult to interpret with regard to treatment outcome. The formalization of PJI consensus criteria has improved treatment standardization in our institution over the past year and we plan to re-evaluate daptomycin performance on more recent cases treated by a smaller group of high volume revision surgeons.

There may be a role for including daptomycin in PMMA spacers, which requires further study. Kuo^33^ reported 100% treatment success when daptomycin was included as 10% weight of a PMMA spacer followed by systemic therapy of 6mg/kg for 2 weeks. No significant side effects related to daptomycin were observed. Finally, the combination of daptomycin with rifampin, which has been linked to improved clearance of staphylococcal biofilm in multiple models of study^34^, should be the target of future formal clinical investigations to determine if a clinical effect on outcomes and prevention of emergence of resistance is observed.

In conclusion, this study demonstrates that daptomycin can be safely utilized for the treatment of staphylococcal PJI, with treatment outcomes which appear similar to commonly used comparator antimicrobials. The treatment success rates overall for both DAIR and two-stage cohorts were comparable but lower than contemporarily reported rates. Although eosinophilic pneumonitis and myotoxicity are rare complications of daptomycin therapy, we identified several cases in our cohort and advise awareness of these unusual entities.

## Figures and Tables

**Table 1:** Univariate analyses of patient characteristics according to surgical treatment and antibiotic therapy

	Debridement and Implant Retention						Two-Stage Exchange				
	No Daptomycin (N=114)		Daptomycin (N=27)				No Daptomycin (N=150)		Daptomcyin (N=50)		
	Mean ± SD/ Count (%)		Mean ± SD/ Count (%)		P-value		Mean ± SD/ Count (%)		Mean ± SD/ Count (%)		P-value
**Age**	63.8 ± 12.5		66.7 ± 8.8		0.168		65.2 ± 12.4		64.4 ± 11.7		0.707
**BMI**	30 ± 6.8		31.1 ±7.5		0.450		29.8 ± 7		31.4 ± 7.8		0.198
**Sex [M:F]**					0.812						0.456
Male	62 (54.4)		14 (51.9)				90 (60.0)		27 (54.0)		
Female	52 (45.6)		13 (48.2)				60 (40.0)		23 (46.0)		
**Acute PJI [Yes:No]**					0.707						-
Yes	42 (36.8)		11 (40.7)				-		-		
No	72 (63.2)		16 (59.3)				-		-		
**Bacterial Species**					0.028*						0.004*
CONS	36 (31.6)		7 (25.9)				65 (43.3)		15 (30.0)		
MRSA	14 (12.3)		9 (33.3)				25 (16.7)		12 (24.0)		
MSSA	64 (56.1)		11 (40.7)				62 (41.3)		21 (42.0)		
**CC Index**					0.502						0.979
0	37 (32.5)		9 (33.3)				54 (36.0)		18 (36.0)		
1-2	50 (43.9)		9 (33.3)				52 (34.7)		18 (36.0)		
3+	27 (23.7)		9 (33.3)				44 (29.3)		14 (28.0)		
**Diabetes**	25 (21.9)		7 (25.9)		0.656		25 (16.7)		11 (22.0)		0.395
**RA**	21 (18.4)		6 (22.2)		0.652		22 (14.7)		5 (10.0)		0.403

BMI: body mass index, CONS: coagulase negative *Staphylococcus*, MRSA: methicillin resistant *Staphylococcus aureus*, MSSA: methicillin resistant *Staphylococcus aureus*, CC Index: Charlson comorbidity index. * denotes p < 0.05.

**Table 2:** Regression outcomes according to surgical treatment

	Odds Ratio	95% CI		P-value
**Debridement and Implant Retention**				
**Age**	1.04	1.00 - 1.08		0.041*
**BMI**	1.04	0.98 - 1.11		0.095
**Male vs Female**	0.69	0.32 - 1.48		0.342
**Daptomycin Use [Yes vs No]**	1.70	0.62 - 4.65		0.301
**Acute vs Non-Acute PJI**	0.61	0.28 - 1.34		0.216
**Bacterial Species**				
CONS vs MRSA	5.58	1.65 - 19.97		0.007*
MSSA vs MRSA	3.68	1.09 - 11.71		0.031*
**CC Index**				
Score of 1-2 versus 0	0.75	0.32 - 1.86		0.517
Score of 3+ versus 0	0.20	0.06 - 0.53		0.003*
			
**Two Stage Exchange**				
**Age**	1.01	0.98 - 1.04		0.482
**BMI**	0.96	0.90 - 1.01		0.114
**Male vs Female**	1.08	0.53 - 2.20		0.838
**Daptomycin Use [Yes vs No]**	0.58	0.27 - 1.26		0.170
**Bacterial Species**				
CONS vs MRSA	1.83	0.7 - 4.81		0.219
MSSA vs MRSA	1.32	0.53 - 3.27		0.556
**CC Index**				
Score of 1-2 versus 0	0.53	0.21 - 1.30		0.164
Score of 3+ versus 0	0.32	0.13 - 0.80		0.014*

BMI: body mass index, CONS: coagulase negative *Staphylococcus*, MRSA: methicillin resistant *Staphylococcus aureus*, MSSA: methicillin resistant *Staphylococcus aureus*, CC Index: Charlson comorbidity index. * denotes p < 0.05.

**Table 3:** Univariate analyses according to surgical treatment and outcome

	Debridement and Implant Retention						Two-Stage Exchange				
	Success (N=74)		Failure (N=67)				Success (N=154)		Failure (N=46)		
	Mean ± SD/ Count		Mean ± SD/ Count		P-value		Mean ± SD/ Count		Mean ± SD/ Count		P-value
**Age**	65.8 ± 11.7		62.8 ± 11.9		0.139		65.2 ± 11.8		64 ± 13.4		0.503
**BMI**	30.9 ± 7.5		29.4 ± 6.1		0.202		29.6 ± 6.1		32.4 ± 9.9		0.154
**Sex [M:F]**	38 : 36		38 : 29		0.523		89 : 63		28 : 20		0.979
**Daptomycin [Yes:No]**	15:59		12 : 55		0.722		34 : 118		6 : 32		0.126
**Acute PJI [Yes:No]**	25:49		28:39		0.327		-		-		-
**Bacterial Species**					0.007*						0.256
CONS	30		13				65		15		
MRSA	7		16				25		12		
MSSA	37		38				62		21		
**CC Index**					0.009*						0.012*
0	27		19				62		10		
1-2	36		23				53		17		
3+	11		25				37		21		

BMI: body mass index, CONS: coagulase negative *Staphylococcus*, MRSA: methicillin resistant *Staphylococcus aureus*, MSSA: methicillin resistant *Staphylococcus aureus*, CC Index: Charlson comorbidity index. * denotes p < 0.05.

## References

[1] Kapadia BH, Berg RA, Daley JA, Fritz J, Bhave A, Mont MA (2016). Periprosthetic joint infection. *Lancet Lond Engl*.

[2] Berend KR, Lombardi AV, Morris MJ, Bergeson AG, Adams JB, Sneller MA (2013). Two-stage treatment of hip periprosthetic joint infection is associated with a high rate of infection control but high mortality. *Clin Orthop*.

[3] Geipel U (2009). Pathogenic organisms in hip joint infections. *Int J Med Sci*.

[4] Drago L, De Vecchi E, Bortolin M, Zagra L, Romanò CL, Cappelletti L (2017). Epidemiology and Antibiotic Resistance of Late Prosthetic Knee and Hip Infections. *J Arthroplasty*.

[5] Ravi S, Zhu M, Luey C, Young SW (2016). Antibiotic resistance in early periprosthetic joint infection. *ANZ J Surg*.

[6] Edelstein AI, Okroj KT, Rogers T, Della Valle CJ, Sporer SM (2018). Nephrotoxicity After the Treatment of Periprosthetic Joint Infection With Antibiotic-Loaded Cement Spacers. *J Arthroplasty*.

[7] Patel RA, Baker HP, Smith SB (2018). Acute Renal Failure due to a Tobramycin and Vancomycin Spacer in Revision Two-Staged Knee Arthroplasty. *Case Rep Nephrol*.

[8] Tótoli EG, Garg S, Salgado HRN (2015). Daptomycin: Physicochemical, Analytical, and Pharmacological Properties. *Ther Drug Monit*.

[9] Oleson FB, Berman CL, Li AP (2004). An evaluation of the P450 inhibition and induction potential of daptomycin in primary human hepatocytes. *Chem Biol Interact*.

[10] Kullar R, McClellan I, Geriak M, Sakoulas G (2014). Efficacy and safety of daptomycin in patients with renal impairment: A multicenter retrospective analysis. *Pharmacotherapy*.

[11] Claeys KC, Zasowski EJ, Casapao AM (2016). Daptomycin Improves Outcomes Regardless of Vancomycin MIC in a Propensity-Matched Analysis of Methicillin-Resistant Staphylococcus aureus Bloodstream Infections. *Antimicrob Agents Chemother*.

[12] Hall Snyder AD, Vidaillac C, Rose W, McRoberts JP, Rybak MJ (2015). Evaluation of High-Dose Daptomycin Versus Vancomycin Alone or Combined with Clarithromycin or Rifampin Against Staphylococcus aureus and S. epidermidis in a Novel In Vitro PK/PD Model of Bacterial Biofilm. *Infect Dis Ther*.

[13] Saleh-Mghir A, Muller-Serieys C, Dinh A, Massias L, Crémieux A-C (2011). Adjunctive rifampin is crucial to optimizing daptomycin efficacy against rabbit prosthetic joint infection due to methicillin-resistant Staphylococcus aureus. *Antimicrob Agents Chemother*.

[14] Garrigós C, Murillo O, Euba G (2010). Efficacy of Usual and High Doses of Daptomycin in Combination with Rifampin versus Alternative Therapies in Experimental Foreign-Body Infection by Methicillin-Resistant Staphylococcus aureus. *Antimicrob Agents Chemother*.

[15] John A-K, Baldoni D, Haschke M (2009). Efficacy of daptomycin in implant-associated infection due to methicillin-resistant Staphylococcus aureus: Importance of combination with rifampin. *Antimicrob Agents Chemother*.

[16] Browne C, Muszbek N, Chapman R (2016). Comparative healthcare-associated costs of methicillin-resistant Staphylococcus aureus bacteraemia-infective endocarditis treated with either daptomycin or vancomycin. *Int J Antimicrob Agents*.

[17] Bhavnani SM, Prakhya A, Hammel JP, Ambrose PG (2009). Cost-Effectiveness of daptomycin versus vancomycin and gentamicin for patients with methicillin- resistant Staphylococcus aureus bacteremia and/or endocarditis. *Clin Infect Dis Off Publ Infect Dis Soc Am*.

[18] Joseph C, Robineau O, Titecat M (2019). Daptomycin versus Vancomycin as Post-Operative Empirical Antibiotic Treatment for Prosthetic Joint Infections: A Case-Control Study. *J Bone Jt Infect*.

[19] Chang Y-J, Lee MS, Lee C-H, Lin P-C, Kuo F-C (2017). Daptomycin treatment in patients with resistant staphylococcal periprosthetic joint infection. *BMC Infect Dis*.

[20] Montange D, Berthier F, Leclerc G (2014). Penetration of daptomycin into bone and synovial fluid in joint replacement. *Antimicrob Agents Chemother*.

[21] Parvizi J, Gehrke T (2014). Definition of Periprosthetic Joint Infection. *J Arthroplasty*.

[22] Quan H, Li B, Couris CM (2011). Updating and validating the Charlson comorbidity index and score for risk adjustment in hospital discharge abstracts using data from 6 countries. *Am J Epidemiol*.

[23] Diaz-Ledezma C, Higuera CA, Parvizi J (2013). Success after treatment of periprosthetic joint infection: A Delphi-based international multidisciplinary consensus. *Clin Orthop*.

[24] Seaton RA, Gonzalez-Ruiz A, Cleveland KO, Couch KA, Pathan R, Hamed K (2016). Real-world daptomycin use across wide geographical regions: Results from a pooled analysis of CORE and EU-CORE. *Ann Clin Microbiol Antimicrob*.

[25] Kim PW, Sorbello AF, Wassel RT, Pham TM, Tonning JM, Nambiar S (2012). Eosinophilic pneumonia in patients treated with daptomycin: Review of the literature and US FDA adverse event reporting system reports. *Drug Saf*.

[26] Arbeit RD, Maki D, Tally FP, Campanaro E, Eisenstein BI, Daptomycin 98-01, 99-01 Investigators (2004). The safety and efficacy of daptomycin for the treatment of complicated skin and skin-structure infections. *Clin Infect Dis Off Publ Infect Dis Soc Am*.

[27] Kostrominova TY, Coleman S, Oleson FB, Faulkner JA, Larkin LM (2010). Effect of daptomycin on primary rat muscle cell cultures in vitro. *In Vitro Cell Dev Biol Anim*.

[28] Hohenegger M (2012). Drug induced rhabdomyolysis. *Curr Opin Pharmacol*.

[29] Odum SM, Fehring TK, Lombardi AV (2011). Irrigation and debridement for periprosthetic infections: Does the organism matter?. *J Arthroplasty*.

[30] Urish KL, Bullock AG, Kreger AM (2018). A Multicenter Study of Irrigation and Debridement in Total Knee Arthroplasty Periprosthetic Joint Infection: Treatment Failure Is High. *J Arthroplasty*.

[31] Löwik CAM, Jutte PC, Tornero E (2018). Predicting Failure in Early Acute Prosthetic Joint Infection Treated With Debridement, Antibiotics, and Implant Retention: External Validation of the KLIC Score. *J Arthroplasty*.

[32] Tertiary care centre adherence to unified guidelines for management of periprosthetic joint infections (2020). A gap analysis. https://www.ncbi.nlm.nih. gov/pmc/articles/PMC5785287/. Accessed February 19.

[33] Kuo F-C, Yen S-H, Peng K-T, Wang J-W, Lee MS (2016). Methicillin-resistant Staphylococcal periprosthetic joint infections can be effectively controlled by systemic and local daptomycin. *BMC Infect Dis*.

[34] Zimmerli W, Sendi P (2019). Role of Rifampin against Staphylococcal Biofilm Infections In Vitro, in Animal Models, and in Orthopedic-Device-Related Infections. *Antimicrob Agents Chemother*.

